# Identification of histone methylation modifiers and their expression patterns during somatic embryogenesis in *Hevea brasiliensis*

**DOI:** 10.1590/1678-4685-GMB-2018-0141

**Published:** 2020-02-17

**Authors:** Hui-Liang Li, Dong Guo, Jia-Hong Zhu, Ying Wang, Shi-Qing Peng

**Affiliations:** 1 Key Laboratory of Tropical Crop Biotechnology, Ministry of Agriculture, Institute of Tropical Bioscience and Biotechnology, Chinese Academy of Tropical Agricultural Sciences, Haikou, China

**Keywords:** Hevea brasiliensis, histone methylation, histone methyltransferase, histone demethylase, somatic embryogenesis

## Abstract

Histone methylation plays a crucial role in various biological processes, from heterochromatin formation to transcriptional regulation. Currently, no information is available regarding histone methylation modifiers in the important rubber-producing plant *He*v*ea brasiliensis*. Here, we identified 47 histone methyltransferase (HMT) genes and 25 histone demethylase (HDM) genes as possible members of the histone methylation modifiers in the rubber tree genome. According to the structural features of HMT and HDM, the HbHMTs were classified into two groups (HbPRMs and HbSDGs), the HbHDMs have two groups (HbLSDs and HbJMJs). Expression patterns were analyzed in five different tissues and at different phases of somatic embryogenesis. *HbSDG10, 21, 25, 33, HbJMJ2, 18, 20* were with high expression at different phases of somatic embryogenesis. *HbSDG10,14, 20, 21, 33* and *HbPRMT4* were expressed highly in anther, *HbSDG14, 20, 21, 22, 23, 33, 35* and *HbPRMT1 HbJMJ7* and *HbLSD1, 2, 3, 4* showed high expression levels in callus. *HbSDG1, 7, 10, 13, 14, 18, 19, 21, 22, 23, 35, HbPRMT1, 8, HbJMJ5, 7, 11, 16, 20* and *HbLSD2, 3, 4* were expressed highly in somatic embryo. *HbSDG10, 21, 25, 33, HbLSD2, 3* were expressed highly in bud of regenerated plant. The analyses reveal that *HbHMTs* and *HbHDMs* exhibit different expression patterns at different phases during somatic embryogenesis, implying that some *HbHMTs* and *HbHDMs* play important roles during somatic embryogenesis. This study provide fundamental information for further studies on histone methylation in *He*v*ea brasiliensis*.

## Introduction

Histone methylation plays an essential role in maintaining genome stability and is also involved in regulating multiple cellular processes (Jenuwein and Allis, 2001; Kouzarides, 2002; Liu *et al.*, 2010). Histone methylation can either repress or activate gene expression (Rice *et al.*, 2003; Liu *et al.*, 2010). The level of histone methylation is dynamically regulated by histone methyltransferases (HMTs) and histone demethylases (HDMs), respectively (Klose and Zhang, 2007; Liu *et al.*, 2010). Histone methylation, occurring at arginine residues or lysine residues, is controlled by protein arginine methyltransferases (PRMTs) and histone lysine methyltransferases (HKMTs) (Jenuwein and Allis, 2001; Litt *et al.*, 2009; Liu *et al.*, 2010). HKMTs, also known as SET-domain group (SDG-like types), and PRMTs have highly conserved SET and PRMT domains involved in methyltransferase catalytic activity (Ng *et al.*, 2007; Liu *et al.*, 2010; Ahmad and Cao, 2012). In contrast with HMTs, HDMs remove the methyl groups from methylated lysine or arginine residues of histones (Tsukada *et al.*, 2006; Chang *et al.*, 2007). Plant HDMs have two major types: lysine-specific demethylase1 (LSD) and jumonjiC (JmjC) domain containing proteins (Shi *et al.*, 2004; Tsukada *et al.*, 2006). LSD removes mono- and di-methyl groups from H3K4 residue depending on flavin adenine dinucleotide as a cofactor, while the JmjC-domain proteins require the presence of a-ketoglutarate and Fe-II cofactors (Shi *et al.*, 2004; Klose and Zhang, 2007). JmjC proteins also remove methyl from H3R2 and H4R3 residues (Chang *et al.*, 2007; Cho *et al.*, 2012). In plants, histone methylation has important roles in cellular processes (Berr *et al.*, 2011), including vegetative growth (Thorstensen *et al.*, 2011; Liu *et al.*, 2017), development (Cartagena *et al.*, 2008; Grini *et al.*, 2009; Cho *et al.*, 2012), circadian cycle (Jones and Harmer, 2011; Lu *et al.*, 2011), flowering process (Gan *et al.*, 2014; Liu *et al.*, 2015), flowering time (Liu *et al.*, 2014, 2016), response to abiotic stress (van Dijk *et al.*, 2010; Shen *et al.*, 2014), disease resistance (Berr *et al.*, 2010; Li *et al.*, 2014), and hormone signaling (Sui *et al.*, 2012; Li J *et al.*, 2015; Zhao *et al.*, 2015).

The rubber tree (*Hevea brasiliensis*) produces natural rubber, which is an important industrial material (Backhaus, 1985). Large scale propagation of rubber tree is achieved by grafting buds onto unselected seedlings (Clément-Demange *et al.*, 2007; Hua *et al.*, 2010). The grafted plants sometimes produce intraclonal heterogeneity for growth and productivity (Chandrashekar *et al.*, 1997; Clément-Demange *et al.*, 2007; Hua *et al.*, 2010). In the early 1980s, novel plantlets, named as self-rooting juvenile clones (SRJCs), developed from internal integuments of immature fruits or anthers of *H. brasiliensis* were obtained through tissue culture (Wang *et al.*, 1980; Carron and Enjalric, 1982). SRJCs showed better performance in growth and rubber yield than those of donor clones (DCs) (Liu *et al.*, 1985; Yang and Mo 1994; Yuan *et al.*, 1998; Chen *et al.*, 2002). The molecular mechanism associated with high yield in SRJCs is not clear. Li *et al.* (2014, 2016) found that there were many differentially expressed genes between SRJCs and DCs, including genes involved in rubber biosynthesis pathway and some genes encoding epigenetic modification enzymes (Li *et al.*, 2014, 2016). The DNA methylation level in rubber tree differs at each periods of somatic embryogenesis (Li HL *et al.*, 2015). Epigenetic modifications may be associated with the regulation of several genes involved in natural rubber biosynthesis, resulting in the higher rubber productivity of SRJCs compared to DCs (Li *et al.*, 2016). Histone methylation is one of the epigenetic modifications (Liu *et al.*, 2010). Up to now, histone methylation modifiers have not been identified in *Hevea brasiliensis*. Using the rubber tree genome data (Rahman *et al.*, 2013; Tang *et al.*, 2016), we investigated histone methylation modifiers through a bioinformatics approach. Here, 47 HMT and 25 HDM genes were identified, and the expression patterns of *HbMTs* and *HbHDMs* were analyzed in different tissues and at different phases of rubber tree somatic embryogenesis. This study should greatly facilitate the functional characterization of those histone methylation in the rubber producing crop species.

## Materials and Methods

### Prediction of HMTs and HDMs in the rubber tree genome

A local rubber tree genome database was established using the rubber tree genome data (Li *et al.*, 2017). The HMTs and HDMs protein sequences from rice and *Arabidopsis thaliana* were used as query sequences (Table S1). A BLAST search was performed to detect HMTs and HDMs, with a BLAST threshold of 1e^-5^. To further verify the reliability of these candidate sequences, the Pfam database (http://pfam.sanger. ac.uk/search) and SMART (http://smart.embl-heidelberg.de/) were used to confirm each candidate HbHMT and HbHDM protein as a member of the HMT and HDM families.

### Motif detection of HbHMTs and HbHDMs

Motif detection of HbHMTs and HbHDMs was performed in http://meme-suite.org/tools/meme (Bailey *et al.*, 2015). All identified motifs were further searched in the InterPro database with Inter-ProScan (Mitchell *et al.*, 2014).

### Multiple sequence alignment and phylogenetic analysis of HbHMTs and HbHDMs

Multiple sequence alignments of HbHMTs and HbHDMs were performed using ClustalX2 (http://www. clustal.org/). A phylogenetic tree of HHbMTs and HbHDMs was constructed by the Maximum Likelihood (ML) method with parameters of bootstrap (1000 replicates). Gene structures were analyzed by comparing the coding sequences of *HbHMTs* and *HbHDMs* with the corresponding genomic sequences using GSDS software (http://gsds.cbi. pku.edu.cn/).

### Plant material

*H. brasiliensis* cultivars RY7-33-97 SRJCs were produced through anthers as explants by tissue culture according to Wang *et al.* (1980) and planted in the Experimental Farm of the Institute of Tropical Bioscience and Biotechnology. Latex (LX), bark (BA), Root (RT), leaf (LF), flower (FL), anther (AN), callus (CA), somatic embryo (SE) and regenerated plant (BRP) were harvested for RNA extraction.

### Expression analysis of *HbHMTs* and *HbHDMs*

Isolation of latex RNA was performed according to Tang *et al.* (2007), and RNA from other tissues was isolated using the RNAprep pure plant kit (TIANGEN, China). Quantitative PCR (qPCR) was performed according to Li *et al.* (2017). qPCR was performed with the primers listed in Table S2. An actin gene (GenBank HQ260674) was used as internal control. Three independent biological replicates were assayed. Gene expression levels were calculated by the 2^-DDCt^ method. A heat map was created using log_2_ based on the average of values from three qPCR data.

### Expression profiles of *HbHMTs* and *HbHDMs* in latex of SRJCs and DCs

The transcriptome data of latex from SRJCs and DCs were obtained from a previously study (Li *et al.*, 2016). The Reads Per Kb per Million Reads Mapped (RPKM) value was used to analyze the expression profiles of *HbHMTs* and *HbHDMs* in latex of SRJCs and DCs*.* A heat map was created using log_2_[RPKM].

### Data archiving statement

Nucleotide sequences of 47 *HMTs* and 25 *HDMs* were deposited with the GenBank XM_021786768.1, XM_021834736.1, XM_021834736.1, XM_021828898.1, XM_021806158.1, XM_021782931.1, XM_021803290.1, XM_021812039.1, XM_021824874.1, XM_021800084.1, XM_021834835.1, XM_021800066.1, XM_021818539.1, XM_021795250.1, XM_021821496.1, XM_021794700.1, XM_021786520.1, XM_021827306.1, XM_021817790.1, XM_021837061.1, XM_021820973.1, XM_021807740.1, XM_021817272.1, XM_021817249.1, XM_021817902.1, XM_021827573.1, XM_021827163.1, XM_021781563.1, XM_021787829.1, XM_021781563.1, XM_021792442.1, XM_021785572.1, XM_021781562.1, XM_021790643.1, XM_021783766.1, XM_021798535.1, XM_021814753.1, XM_021779747.1, XM_021789206.1, XM_021802565.1, XM_021802564.1, XM_021790468.1, XM_021815283.1, XM_021790438.1, XM_021789001.1, XM_021795575.1, XM_021812048.1, XM_021790481.1, XM_021835117.1, XM_021784706.1, XM_021803998.1, XM_021789490.1, XM_021750304.1, XM_021816041.1, XM_021829524.1, XM_021790903.1, XM_021781966.1, XM_021821775.1, XM_021809017.1, XM_021832519.1, XM_021806151.1, XM_021802187.1, XM_021811847.1, XM_021807326.1, XM_021800564.1, XM_021794618.1, XM_021780908.1, XM_021810275.1, XM_021813550.1, XM_021810520.1, XM_021794939.1, XM_021825435.1.

## Results

### Identification of *HbHMTs* and *HbHDMs*

A total of 72 putative genes, including 47 *HbHMTs* and 25 *HbHDMs*, were identified in *Hevea brasiliensis*. *HbHMT*s were designated as *HbSDG1 - HbSDG38*, and *HbPRM1 - HbPRM9. HbHDMs* were named as *HbJMJ1 - HbJMJ20,* and *HbLSD1 - HbLSD5.* The open reading frames of the 72 predicted genes ranged from 822 bp (*HbSDG35*) to 4353 bp (*HbLSD5*) in length (Table S3). The number of exons of the 72 predicted genes ranged from 1 (*HbSDG3, 4, 7,18, 30* and *HbLSD3*) to 28 (*HbJMJ6*).

### The conserved domains and phylogenetic analysis of HbHMTs and HbHDMs

**Figure 1:**
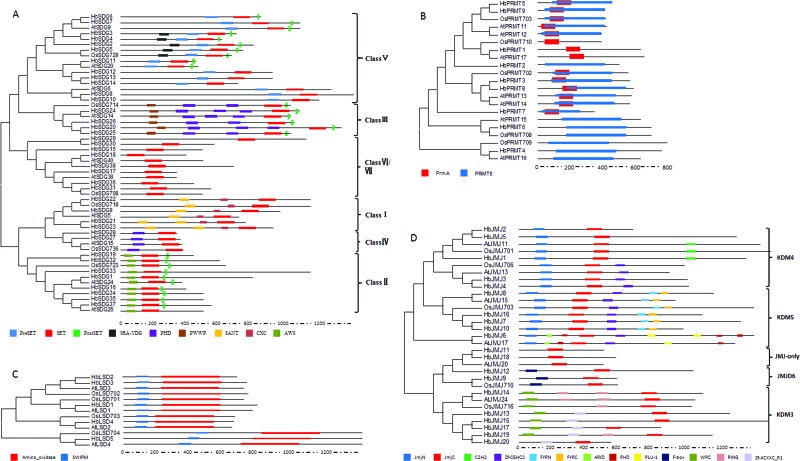
The conserved domains and phylogenetic analysis of HbHMTs and HbHDMs. (A) The conserved domains and phylogenetic analysi**s** of HbSDGs. (B) The conserved domains and phylogenetic analysis of HbPRMTs. (C) The conserved domains and phylogenetic analysis of HbLSDs. (D) The conserved domains and phylogenetic analysis of HbJMJs. The accession numbers of HMTs and HDMs from *Arabidopsis* and rice employed for the phylogenetic tree analysis are given in Table S1.

The putative HbHMTs were classified as 9 HbPRMs, and 38 HbSDGs. The HbHDMs were 5 HbLSDs and 20 HbJMJs, according to the previous study in *Solanum lycopersicum* and *Citrus sinensis* (Aiese Cigliano *et al.*, 2013; Xu *et al.*, 2015). For HbHMTs, all of the 38 HbSDGs were characterized by a conserved SET domain (PF00856) and were grouped into seven classes. In detail, HbSDG9, 15, 21, 22, and 23, which have a conserved SANT domain (SM00717) and CXC domain (SM001114), belong to class I. Class II consisted of seven HbSDGs, including HbSDG1, 16, 19, 32-34, 37, and contain a conserved AWS domain (SM00570) and Post-SET domain (SM00508), respectively. Class III was comprised of four members (HbSDG 20, 24, 25, 26), which have a Post-SET domain (SM00508), PHD domain (PF00628), and a PWWP domain (SM00293). Class IV included HbSDG27 and 28, which have an N-terminal PHD domain. Nine HbSDGs (HbSDG2-8, 10, 11) belonged to class V and contained a Pre-SET domain (PF05033), Post-SET domain (SM00508), and a SRA-YDG domain (IPR003105). Eight HbSDGs belonged to class VI /VII (Figure 1A). With regard to HbPRMTs, nine predicted HbPRMs proteins were characterized by a PRMT5 domain (PF05185) and categorized into two classes. HbPRMT 1, 3-5, 7-9 belonged to class I, which contains a PrmA domain (PF06325), and HbPRMT4 and 6 were clustered into class II (Figure 1B). For HbHDMs, all of the five HbLSDs have a conserved domain and an amino oxidase domain (PF01593) and SWIRM (PF04433) (Figure 1C). All of the 20 HbJMJs have a conserved JmjC domain (PF02373). The JMJ family is divided into five classes, including JMJ-only, KDM3, KDM4, KDM5, and JMJD6, according to the previous study by Lu *et al.* (2011). The JMJ-only class consisted of HbJMJ11 and 18, which only contain the conserved JmjC domain. KDM3 class included seven members (HbJMJ3, 14-15, 17, 19 and HbJMJ20), characterized by a ring finger domain (SM000184). The KDM4 group HbJMJs were classified into two main subgroups. Subgroup I contains a ZnF_C2H2 domain (SM000355), while subgroup II has a zf-C5HC2 domain (PF02928) at the C-terminus. The class KDM5 comprises five members (HbJMJ6-8, 10, 16) and is characterized by a JmjN domain (PF02375) and zf-C5HC2 domain (PF02928), respectively. Additionally, two members (HbJMJ9 and HbJMJ12) belonged to the JMJD6 class, which contains an N-terminal F-box domain (PF00646) (Figure 1D).

### Expression of *HbHMTs* and *HbHDMs*

**Figure 2:**
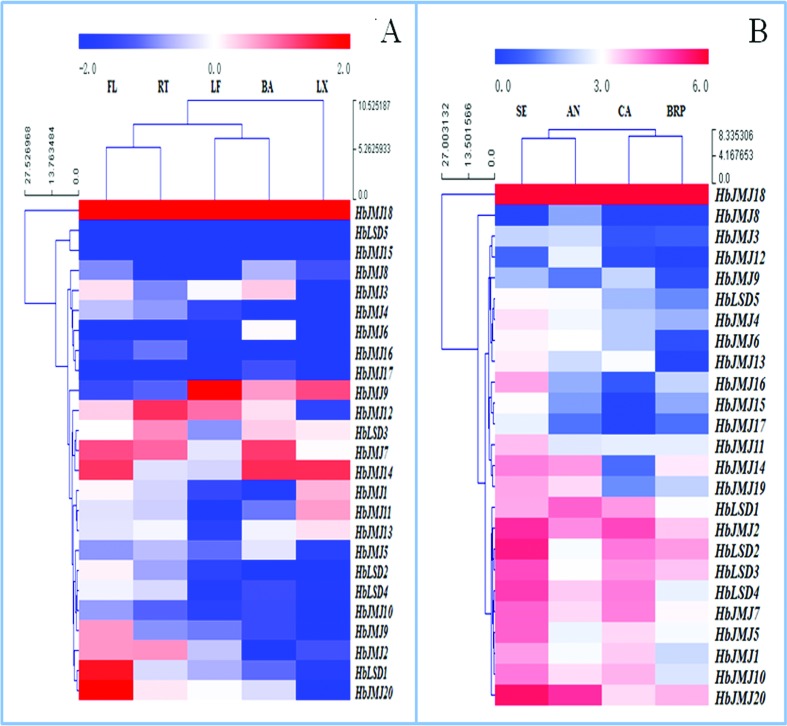
The expression of *HbHMTs* in different tissues (A) and during somatic embryogenesis of rubber tree (B). The heat maps were created using log_2_ based values from three replicates of real-time RT-PCR data. The scale represents the relative signal intensity values. RT, Root; BA, bark; LF, leaf; FL, flowers; LX, latex; AN, Anther; CA, callus; SE, somatic embryo, BRP, bud of regenerated plant.

**Figure 3:**
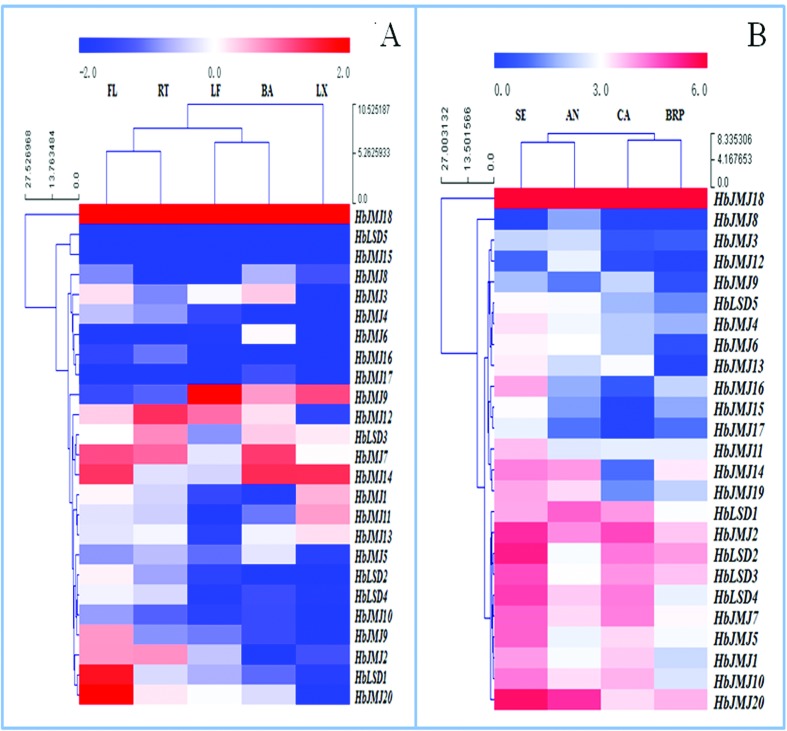
The expression of *HbHDMs* in different tissues (A) and during somatic embryogenesis of rubber tree (B)**.** The heat maps were created using log_2_ based values from three replicates of real-time data. The scale represents the relative signal intensity values. RT, Root; BA, bark; LF, leaf; FL, flowers; LX, latex; AN, Anther; CA, callus; SE, somatic embryo, BRP, bud of regenerated plant.

The expression of *HbHMTs* and *HbHDMs* was investigated in LX, BA, RT, LF, and FL. As shown in Figure 2A and Figure 3A, all these genes were differentially expressed either in terms of their expression patterns or their transcript level. Among the *HbHMTs, HbPRMT18* and *HbSDG25* were highly expressed in all tested tissues, while *HbSDG17, 26, 36,* and *38* had low expression in five tested tissues. *HbPRMT1, 3, 7* and *HbSDG7, 11* showed a high expression level in LX, while *HbPRMT4* and *HbSDG1, 12, 35* were highly expressed in LF. *HbPRMT4* and *HbSDG1, 12,36* had high expression in FL. Some *HbHMTs*, such as *HbPRMT7* and *HbSDG12, 21* were highly expressed in RT. *HbPRMT1* and *HbSDG1, 33* showed a high expression level in BA (Figure 2A). Among the *HbHDMs, HbJMJ18* was highly expression in all tested tissues, while *HbJMJ15* and *HbLSD5* had low expression in five tested tissues. *HbJMJ1, 14* showed a high expression level in LX, while *HbJMJ9, 12* were expressed highly in LF. *HbJMJ7, 14,18* and *HbLSD1* had high expression in FL. *HbJMJ7, 12* and *HbLSD3* were highly expressed in RT. *HbJMJ7, 14* showed a high expression level in BA (Figure 3A).

### The expression of *HbHMTs* and *HbHDMs* at different phases of somatic embryogenesis

An expression analysis of *HbHMTs* and *HbHDMs* was performed to further the understanding of the mechanisms involved in rubber tree somatic embryogenesis. Most of the *HbHMTs* and *HbHDMs* had differential expression profiles during somatic embryogenesis. Among the *HbHMTs*, *HbSDG10, 21, 25, 33* showed high expression at different phases of somatic embryogenesis, while *HbSDG4, 9, 16, 17, 19, 26, 27, 29* and *HbPRMT3, 6, 7, 12* were with low expression at different phases of somatic embryogenesis. *HbSDG10,14, 20, 21, 33* and *HbPRMT4* were expressed highly in AN, *HbSDG14, 20, 21, 22, 23, 33, 35* and *HbPRMT1* showed a high expression level in CA. *HbSDG1, 7, 10, 13, 14, 18, 19, 21, 22, 23, 35,* and *HbPRMT1, 8* were expressed highly in SE. *HbSDG10, 21, 25, 33* were expressed highly in BRP (Figure 2B). Among the *HbHDMs*, *HbJMJ2, 18, 20* were with high expression at different phases of somatic embryogenesis, while *HbJMJ3, 8, 9, 12, 17* and *HbLSD5* were with low expression at different phases of somatic embryogenesis. Additionally, *HbJMJ7* and *HbLSD1, 2, 3, 4,* showed a high expression level in CA *.HbLSD2, 3* were highly expressed in BRP. *HbJMJ2,14, 20* and *HbLSD1* were highly expressed in AN. *HbJMJ5, 7, 11, 16, 20,* and *HbLSD2, 3, 4* were highly expressed in SE. *HbLSD2, 3* showed a high expression level in BRP (Figure 3B).

### Expression profiles of *HbHMTs* and *HbHDMs* in latex compatomg SRJCs and DCs

**Figure 4:**
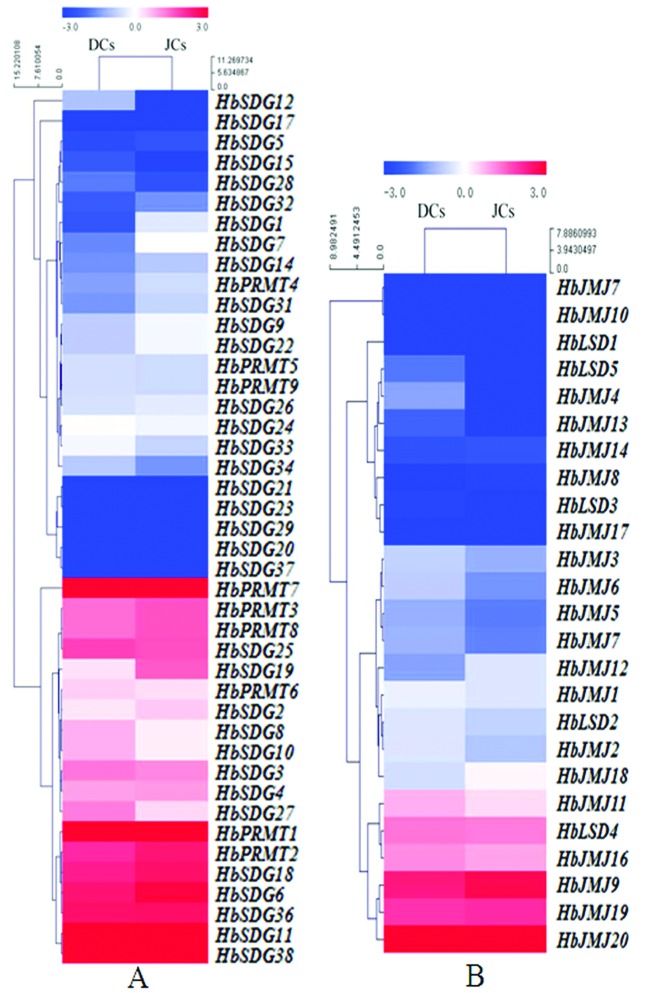
Expression profiles of *HbHMTs* and *HbHDMs* in latex. **(**A) Heatmap of *HbHMTs.* (B) Heatmap of *HbHDMs.* The heatmaps were created using log_2_ based RPKM values from the latex transcriptome data of SRJCs and its DCs. The values in red and blue indicate log_2_[RPKM] fold increases and decreases, respectively.

The expression patterns of *HbHMTs* and *HbHDMs* in latex were analyzed comparing SRJCs and its DCs. Eleven *HbHMTs* (*HbSDG 1, 7-10, 19, 22, 27, 33, 34* and *HbPRMT6*) and five *HbHDMs* (*HbJMJ3, 4, 6, 12, 18* and *HbLSD5)* had differential expression profiles in latex between SRJCs and DCs. Among these 11 differentially expressed *HbHMTs, HbSDG1, 7, 9, 19, 22* were up-regulated in SRJCs, while *HbSDG8, 10, 27, 33, 34* and *HbPRMT6* were up-regulated in DCs (Figure 4A).Among the five *HbHDMs* differentially expressed in latex between SRJCs and DCs, *HbJMJ3, 6, 12* were up-regulated in SRJCs, while *HbJMJ 4, 18* and *HbLSD5* were up-regulated in DCs (Figure 4B).

## Discussion

Plant regeneration via somatic embryogenesis provokes many epigenetics changes including histone modification and DNA methylation (Yakovlev *et al.*, 2016). Histone modification is dynamically regulated during somatic embryogenesis (Pfluger and Wagner, 2007; Eichten *et al.*, 2014; De-la-Peña *et al.*, 2015). The dynamic activity in the modification of histones leads to the modulation of gene expression involved in somatic embryogenesis (Nic-Can *et al.*, 2013). For example, the levels of the histone repressive marks H3K9me2 and H3K27me3 decrease in *C. canephora* during the early events of somatic embryogenesis, and these events were correlated with the beginning of the expression of genes involved in the somatic embryogenesis process. This indicates that the histone modification regulated changes in transcriptional program of the somatic cells before and during the development of somatic embryos (Nic-Can *et al.*, 2013). The H3K9me2 mark has also been involved in embryo cell differentiation and heterochromatization events during microspore embryogenesis in *B. napus* (Rodríguez-Sanz *et al.*, 2014). In addition, the expression patterns of several genes related to histone modification have been studied during the somatic embryogenesis process of *Q. suber* (Pérez *et al.*, 2015). It was found that *QsHDA19* decreases as soon as the callus begins its differentiation, followed by a steady increase from immature cotyledonary embryo to an embryo with fully differentiated cotyledons. On the other hand, a transient decrease in *QsHDA6*, *QsPICKLE*, and *QsVAL1* gene expression was observed at the transition from callus to the end of the mature embryo.

Histone methylation is generally associated with regulating dynamic changes from heterochromatin formation to transcriptional regulation (Kouzarides, 2002). The methylation of histones is involved in repressing or activating gene expression (Lusser *et al.*, 2002). The methylation and demethylation of histones modifications was shown to be associated the with chromatin state in modulating plant growth and development (Berr *et al.*, 2011; Xu *et al.*, 2015). Here, histone methylation modifiers involved in histone methylation/demethylation have been identified in *Hevea brasiliensis*. Four gene groups (*HbSDG*, *HbPRM*, *HbLSD,* and *HbJMJ*) containing 72 genes were characterized in *Hevea brasiliensis*. The numbers of these groups in *Hevea brasiliensis* are close to those in *Citrus sinensis* and *Arabidopsis*. *HbHMTs* and *HbHDMs* have 47 and 25 members, respectively, and the corresponding *AtHMTs* and *AtHDMs* families contain 48 and 25 members (Gu *et al.*, 2016), *CsHMTs* and *CsHDMs* families contain 47 and 23 members (Xu *et al.*, 2015).

The expressions profiles of all *HbHMTs* and *HbHDMs* were analyzed in rubber tree tissues. The differential expression of *HbHMTs* and *HbHDMs* in different rubber tree tissues indicates a broad role for some *HbHMTs* and *HbHDMs*. The highly expressed *HbHMTs* and *HbHDMs* or differentially expressed *HbHMTs* and *HbHDMs* in different tissues may play an important role in rubber tree growth and development. It will be of interest to elucidate the functions of *HbHMTs* and *HbHDMs*.

During plant somatic embryogenesis, plant growth regulators may contribute to induce epigenetic modifications (Miguel and Marum, 2011; Machczynska *et al.*, 2015). The modification of histones leads to the modulation of the expression of genes involved in the somatic embryogenesis process (De-la-Peña *et al.*, 2015). In this study, several genes encoding histone methylation modifiers were shown to be differentially expressed in latex comparing SRJCs and DCs, including eleven *HbHMTs* (*HbSDG 1, 7-10, 19, 22, 27, 33, 34* and *HbPRMT6*) and five *HbHDMs* (*HbJMJ3, 4, 6, 12, 18* and *HbLSD5)*. Latex is the cytoplasm of laticifer cells in *H. brasiliensis* (Chow *et al.*, 2007). Laticifers in the bark of the rubber tree are specific for rubber biosynthesis (Hao and Wu, 2000). Histone methylation modifications, thus, may provide new insights into the molecular mechanism associated with high yield in SRJCs.

## Supplementary material

The following online material is available for this article:

Table S1 The accession number of HMTs and HDMs from *Arabidopsis* and rice.

Table S2 Overview of the primer sequences for real time PCR.

Table S3 Overview of the detected histone modification genes in *Hevea brasiliensis.*
