# Supplementary material for: Identification of histone methylation modifiers and their expression patterns during somatic embryogenesis in Hevea brasiliensis
Source: Genet Mol Biol. 2020 Feb 17;43(1):e20180141. doi: 10.1590/1678-4685-GMB-2018-0141 (PMC7229888; doi:10.1590/1678-4685-GMB-2018-0141)
Supplement: Supplementary file 1 [file 1415-4757-GMB-43-1-e20180141-20200116-suppl1.pdf]

# Supplementary Material to “Identification of histone methylation modifiers and their expression patterns during somatic embryogenesis in *Hevea brasiliensis*”

**Table S1** - List of the histone modifiers from *Aradopsis thaliana and Oryza sativa* used in this study

| NO. | ChromDB ID | Locus | Organism |
|---|---|---|---|
| 1 | LSD1 | At3g10390 | *Arabidopsis thaliana* |
| 2 | LSD2 | At3g13682 | *Arabidopsis thaliana* |
| 3 | LSD3 | At1g62830 | *Arabidopsis thaliana* |
| 4 | LSD4 | At4g16310 | *Arabidopsis thaliana* |
| 5 | LSD701 | LOC_Os04g47270 | *Oryza sativa* |
| 6 | LSD702 | LOC_Os02g51880 | *Oryza sativa* |
| 7 | LSD703 | LOC_Os08g04780 | *Oryza sativa* |
| 8 | LSD704 | LOC_Os10g38850 | *Oryza sativa* |
| 9 | JMJ11 | At5g04240 | *Arabidopsis thaliana* |
| 10 | JMJ13 | At5g46910 | *Arabidopsis thaliana* |
| 11 | JMJ15 | At2g34880 | *Arabidopsis thaliana* |
| 12 | JMJ17 | At1g63490 | *Arabidopsis thaliana* |
| 13 | JMJ20 | At5g63080 | *Arabidopsis thaliana* |
| 14 | JMJ24 | At1g09060 | *Arabidopsis thaliana* |
| 15 | JMJ701 | LOC_Os03g05680 | *Oryza sativa* |
| 16 | JMJ703 | LOC_Os05g10770 | *Oryza sativa* |
| 17 | JMJ706 | LOC_Os10g42690 | *Oryza sativa* |
| 18 | JMJ710 | LOC_Os11g36450 | *Oryza sativa* |
| 19 | JMJ716 | LOC_Os03g22540 | *Oryza sativa* |
| 20 | PRMT6 | At3G20020 | *Arabidopsis thaliana* |
| 21 | PRMT10 | At1G04870 | *Arabidopsis thaliana* |
| 22 | PRMT11 | At4g29510 | *Arabidopsis thaliana* |
| 23 | PRMT12 | At2g19670 | *Arabidopsis thaliana* |
| 24 | PRMT13 | At3g06930 | *Arabidopsis thaliana* |
| 25 | PRMT14 | At5g49020 | *Arabidopsis thaliana* |
| 26 | PRMT15 | At4g31120 | *Arabidopsis thaliana* |
| 27 | PRMT16 | At4g16570 | *Arabidopsis thaliana* |
| 28 | PRMT17 | At3g12270 | *Arabidopsis thaliana* |
| 29 | PRMT702 | LOC_Os07g47500 | *Oryza sativa* |
| 30 | PRMT703 | LOC_Os09g19560 | *Oryza sativa* |
| 31 | PRMT705 | LOC_Os06g05090 | *Oryza sativa* |
| 32 | PRMT706 | LOC_Os04g58060 | *Oryza sativa* |
| 33 | PRMT708 | LOC_Os02g04660 | *Oryza sativa* |
| 34 | PRMT709 | LOC_Os06g01640 | *Oryza sativa* |
| 35 | PRMT710 | LOC_Os07g44640 | *Oryza sativa* |
| 36 | SDG1 | At2g23380 | *Arabidopsis thaliana* |
| 37 | SDG2 | At4g15180 | *Arabidopsis thaliana* |
| 38 | SDG3 | At2g33290 | *Arabidopsis thaliana* |
| 39 | SDG4 | At4g30860 | *Arabidopsis thaliana* |
| 40 | SDG5 | At1g02580 | *Arabidopsis thaliana* |
| 41 | SDG6 | At2g23740 | *Arabidopsis thaliana* |
| 42 | SDG7 | At2g44150 | *Arabidopsis thaliana* |
| 43 | SDG8 | At1g77300 | *Arabidopsis thaliana* |
| 44 | SDG9 | At2g35160 | *Arabidopsis thaliana* |
| 45 | SDG10 | At4g02020 | *Arabidopsis thaliana* |
| 46 | SDG11 | At2g05900 | *Arabidopsis thaliana* |
| 47 | SDG13 | At1g04050 | *Arabidopsis thaliana* |
| 48 | SDG14 | At3g61740 | *Arabidopsis thaliana* |
| 49 | SDG15 | At5g09790 | *Arabidopsis thaliana* |
| 50 | SDG16 | At4g27910 | *Arabidopsis thaliana* |
| 51 | SDG17 | At1g17770 | *Arabidopsis thaliana* |
| 52 | SDG18 | At5g43990 | *Arabidopsis thaliana* |
| 53 | SDG19 | At1g73100 | *Arabidopsis thaliana* |
| 54 | SDG20 | At3g03750 | *Arabidopsis thaliana* |
| 55 | SDG21 | At2g24740 | *Arabidopsis thaliana* |
| 56 | SDG22 | At4g13460 | *Arabidopsis thaliana* |
| 57 | SDG23 | At2g22740 | *Arabidopsis thaliana* |
| 58 | SDG24 | At3g59960 | *Arabidopsis thaliana* |

| NO. | ChromDB ID | Locus | Organism |
|---|---|---|---|
| 59 | SDG25 | At5g42400 | *Arabidopsis thaliana* |
| 60 | SDG26 | At1g76710 | *Arabidopsis thaliana* |
| 61 | SDG27 | At2g31650 | *Arabidopsis thaliana* |
| 62 | SDG29 | At5g53430 | *Arabidopsis thaliana* |
| 63 | SDG30 | At1g05830 | *Arabidopsis thaliana* |
| 64 | SDG31 | At3g04380 | *Arabidopsis thaliana* |
| 65 | SDG32 | At5g04940 | *Arabidopsis thaliana* |
| 66 | SDG33 | At5g13960 | *Arabidopsis thaliana* |
| 67 | SDG34 | At5g24330 | *Arabidopsis thaliana* |
| 68 | SDG35 | At1g26760 | *Arabidopsis thaliana* |
| 69 | SDG36 | At3g21820 | *Arabidopsis thaliana* |
| 70 | SDG37 | At2g17900 | *Arabidopsis thaliana* |
| 71 | SDG38 | At5g06620 | *Arabidopsis thaliana* |
| 72 | SDG39 | At2g19640 | *Arabidopsis thaliana* |
| 73 | SDG40 | At5g17240 | *Arabidopsis thaliana* |
| 74 | SDG41 | At1g43245 | *Arabidopsis thaliana* |
| 75 | SDG42 | At1g01920 | *Arabidopsis thaliana* |
| 76 | SDG43 | At1g14030 | *Arabidopsis thaliana* |
| 77 | SDG701 | LOC_Os08g08210 | *Oryza sativa* |
| 78 | SDG703 | LOC_Os04g45990 | *Oryza sativa* |
| 79 | SDG704 | LOC_Os11g38900 | *Oryza sativa* |
| 80 | SDG705 | LOC_Os01g46700 | *Oryza sativa* |
| 81 | SDG706 | LOC_Os02g47900 | *Oryza sativa* |
| 82 | SDG707 | LOC_Os08g34370 | *Oryza sativa* |
| 83 | SDG708 | LOC_Os04g34980 | *Oryza sativa* |
| 84 | SDG709 | LOC_Os01g59620 | *Oryza sativa* |
| 85 | SDG710 | LOC_Os08g30910 | *Oryza sativa* |
| 86 | SDG711 | LOC_Os06g16390 | *Oryza sativa* |
| 87 | SDG712 | LOC_Os02g40770 | *Oryza sativa* |
| 88 | SDG713 | LOC_Os03g20430 | *Oryza sativa* |
| 89 | SDG714 | LOC_Os01g70220 | *Oryza sativa* |
| 90 | SDG715 | LOC_Os08g45130 | *Oryza sativa* |
| 91 | SDG716 | LOC_Os03g49730 | *Oryza sativa* |
| 92 | SDG717 | LOC_Os12g41900 | *Oryza sativa* |
| 93 | SDG718 | LOC_Os03g19480 | *Oryza sativa* |
| 94 | SDG720 | LOC_Os01g73460 | *Oryza sativa* |
| 95 | SDG721 | LOC_Os01g11950 | *Oryza sativa* |
| 96 | SDG722 | LOC_Os04g53700 | *Oryza sativa* |
| 97 | SDG723 | LOC_Os09g04890 | *Oryza sativa* |
| 98 | SDG724 | LOC_Os09g13740 | *Oryza sativa* |
| 99 | SDG725 | LOC_Os02g34850 | *Oryza sativa* |
| 100 | SDG726 | LOC_Os07g25450 | *Oryza sativa* |
| 101 | SDG727 | LOC_Os09g19830 | *Oryza sativa* |
| 102 | SDG728 | LOC_Os05g41170 | *Oryza sativa* |
| 103 | SDG729 | LOC_Os01g56540 | *Oryza sativa* |
| 104 | SDG730 | LOC_Os02g03030 | *Oryza sativa* |
