# Supplementary material for: Identification of histone methylation modifiers and their expression patterns during somatic embryogenesis in Hevea brasiliensis
Source: Genet Mol Biol. 2020 Feb 17;43(1):e20180141. doi: 10.1590/1678-4685-GMB-2018-0141 (PMC7229888; doi:10.1590/1678-4685-GMB-2018-0141)
Supplement: Supplementary file 2 [file 1415-4757-GMB-43-1-e20180141-20200116-suppl2.pdf]

Supplementary Material to "Identification of histone methylation modifiers and their expression patterns during somatic embryogenesis in *Hevea brasiliensis*"

Table S2 - Overview of the primer sequences for real time PCR

No.	Name	Accession number	Primer sequences for real time PCR (nucleic sequences from 5' to 3')	
		(GenBank)	forward	reverse
1	HbLSD1	XM_021786768.1	GTTTGAGGGAAGCTGCAAATATAG	AGAAGGGAAGCACAGTTATGAG
2	HbLSD2	XM_021834736.1	GACATTCGGGACCTTCTCTATTC	AGACATAGCGAGCAAGAATCC
3	HbLSD3	XM_021834736.1	GAGGCAGGCCATTGAACTAA	CATCAGAGAAGCCTTTCCTACC
4	HbLSD4	XM_021828898.1	GAATAGCAGTAAGCGTGGAGAG	CTCCAGCAACCAATGCAATAAG
5	HbLSD5	XM_021806158.1	CAGGGACTGGTCTGTTGATTT	GAATGGCTCCGCTGAGATAAG
6	HbJMJ1	XM_021782931.1	CACTAAGGAGGGTTCACAGAAG	ATTCCGCTGCAGAGAACTATT
7	HbJMJ2	XM_021803290.1	GGTATGCTGTTCAGTTGGTTTG	CAAGTCTTTGGAGAGCCAGTAT
8	HbJMJ3	XM_021812039.1	TAAGTTCCCGCATGAGTTGG	TGCTCTCTGACCCTTGAAATG
9	HbJMJ4	XM_021824874.1	GAGATTGGATAGATTCCAGCATGAG	TGATGGGCCTCGGACTTTA
10	HbJMJ5	XM_021800084.1	GATTGGGTGACTAGGGATGATG	GGGTCTGGATTAGGCTGATTT
11	HbJMJ6	XM_021834835.1	CTATGTTTGCGCTGCATGTG	CTGTGTGCTGTGGAGAAAGT
12	HbJMJ7	XM_021800066.1	GGTCAGATTCCACAACCTTCT	TCCCAGGAGTCCCTCTATTATC
13	HbJMJ8	XM_021818539.1	CAGCCTTTGCTGAGTGTTAATG	TTGGGATCAAGAGCCTCAATAG
14	HbJMJ9	XM_021795250.1	GGAAGAATCTGTTGCCATTACAC	CACGGTCTTGTGTTCCAGATA
15	HbJMJ10	XM_021821496.1	CCTTCTCAACCCGAAGAAAGT	ACTCCCTCAGGTAGGAGATTAC
16	HbJMJ11	XM_021794700.1	GGACTTGCTTTGGAGAGACTAC	TTGCGTTGACAGACACCTT
17	HbJMJ12	XM_021786520.1	CATGGAGTCCCAGCTACATTC	AGAAGACTGGAGTCACCTCTAA
18	HbJMJ13	XM_021827306.1	TGTTGGAGAAGCTGTCATCAT	AGGCGAGACAAAGTCCAATAC
19	HbJMJ14	XM_021817790.1	CTGCTAAATTGGCTGAGGAAATAC	GGCTGAACTAGCAGCATACA
20	HbJMJ15	XM_021837061.1	ATGGTGTTGTCCACCCTATTC	CTCAACTCCAAACTCCTCCTTC
21	HbJMJ16	XM_021820973.1	GCAATCACAGAGGATGGACAA	CACAGCAGAAAGGTGCAAATC
22	HbJMJ17	XM_021807740.1	TGATCAGCTAAGAGGTGCTAGA	GGAGACATCCTCCCTCCTAAA
23	HbJMJ18	XM_021817272.1	GAAGGGAGATTGAGGTGGTTT	CCTCTAACCCAATGTGCTCTAC
24	HbJMJ19	XM_021817249.1	TGAACCTTGCTGTCCGATTAC	GCTCTTGAGCACAGCCATAA
25	HbJMJ20	XM_021817902.1	CCTTGCTGTCCGATTACCTAAG	GTAACAGAATCTCCACGTCCAA
26	HbPRMT1	XM_021827573.1	CATTCCCTGCTGCAACAATTC	CATCAGGGCCAACACTAACA
27	HbPRMT2	XM_021827163.1	TGCACTTGTGCTGTCTACTG	GTTGATCTTGCTCCACTTCTACT
28	HbPRMT3	XM_021781563.1	GCTACTCCGCAGCACTTTATC	TGAGATGGTGGCTGTATCAAATC
29	HbPRMT4	XM_021787829.1	GTGCCATGGATTTGTGCTATG	CCTTGCTTCCAGTATCTCTCATC
30	HbPRMT5	XM_021781563.1	CTGCCAGCTACTCAAGACTATG	GCTACCAACGCATGAATGTAATC
31	HbPRMT6	XM_021792442.1	GCTCCTTCTCAACCTGTCTTT	GCATCATTCGCAATCTCAAACT
32	HbPRMT7	XM_021785572.1	CTCCTGTTCGCATCAGTGAA	GTCTGATCTCACAGCCAATCTC
33	HbPRMT8	XM_021781562.1	GGTGCTGAACAAGGAGGAATA	GCTTGTGGCTGAGACATTCTA
34	HbPRMT9	XM_021790643.1	TGTGAAAGGGAAGCCCTAAC	GCTGCCCATTCAATGCATATT
35	HbSDG1	XM_021783766.1	GGATACCCACAGGAAACAGTAA	GCCTATGGAGATTGATGGGTAG
36	HbSDG2	XM_021798535.1	GCTGGATCAGTGGATTGATGA	ACCCATAACTCATTGGAGAACC
37	HbSDG3	XM_021814753.1	GTGCTTGTGCAGTTTGTGTTAT	TCAAGGCTAAGTTCCCTCAAAG
38	HbSDG4	XM_021779747.1	ATGTGTCGAGAATGAGGAATGT	CATGAGGTGAGGGAACATCAA
39	HbSDG5	XM_021789206.1	TGACTTTGCTGTTGTGATTTCC	CTATCAAGAGCGTAGCCATAGTC
40	HbSDG6	XM_021802565.1	CACCATTGATGTAGCAAAGTATGG	GGAATTCTCTTGTCCTCGTGAT
41	HbSDG7	XM_021802564.1	GTTCTCTCTGGGATGGACTTTC	TGAAGCAACTCTCCTCCATAAC
42	HbSDG8	XM_021790468.1	GGAGGAGGGTTACCTTGTTTATG	GACAGCCCATCCCTTATTCTTT

No.	Name	Accession number	Primer sequences for real time PCR (nucleic sequences from 5' to 3')	
		(GenBank)	forward	reverse
43	HbSDG9	XM_021815283.1	CAGTCCTCTAAGCAGTACACAC	ACTCTTTGAGCACCCACAATA
44	HbSDG10	XM_021790438.1	TAGATGAGCAGGAGGCAAATG	ACCTGTCCTTCAATCAATCTACTC
45	HbSDG11	XM_021789001.1	GTGAGAGAGCATCTTCCATCAG	GACAGATTCCCGCCATTACA
46	HbSDG12	XM_021795575.1	CAGCCCGGGTTCTCATTATATC	CAAGATTCGCTTCCACAACAAC
47	HbSDG13	XM_021812048.1	GGAAGAGCTGACTTGGGATTAT	AGAACTTGCTGCCACATAGG
48	HbSDG14	XM_021790481.1	CAGGAACATGAAACGTCCAAATAG	AGCTGGATTGTCTGAGATGAAG
49	HbSDG15	XM_021835117.1	CAACCTCAACTGAATTGGCTATC	TTTGCCAGCAATGACTTTACC
50	HbSDG16	XM_021784706.1	GTGTTTGCTGCTCGTTCAATAA	CACTTCACCTCTGGTCCAAAT
51	HbSDG17	XM_021803998.1	ACAATCACGACTGTGATCCTAAT	GAGCTCTTCATCCGCTTCAATA
52	HbSDG18	XM_021789490.1	CCGAACCTAATGGTGGAGAAG	CGAAGGCAAAGGAGCTAGAG
53	HbSDG19	XM_021750304.1	GCACGAACATTGCCTACTTG	CGAGCATCTGTTGGCATTCT
54	HbSDG20	XM_021816041.1	TACGAAGCATGGGGGAATTG	AGTGATTTCTTCCCCAGCAG
55	HbSDG21	XM_021829524.1	GCAGTGATGACACACTAAGGA	CACCCTGTTCCTCCCAAAAA
56	HbSDG22	XM_021790903.1	AGCCACCTAAACGAGGAGAT	GTTCTGAATGCGCACTCTCT
57	HbSDG23	XM_021781966.1	GATCACCGTGTGGGCATATT	CCAGATGATGCTTCAGGCTT
58	HbSDG24	XM_021821775.1	ACCACTCATGTATGCCGAAC	TGTTAGCTCATCACCAGCAG
59	HbSDG25	XM_021809017.1	CGTGTATGCCCAACTGCTAT	CGTTAGCTCATCCCCTTCAG
60	HbSDG26	XM_021832519.1	AGGGGAATGGAACTTCCTGA	TATTGGCTCCTTTTCAGCCC
61	HbSDG27	XM_021806151.1	GATCCACAGGAAGGGTTCAC	CCATCATCATTTTCCCGGCT
62	HbSDG28	XM_021802187.1	CTGGCCCAGTCAAGATTACC	AAGAACCCTAGGAAGCACCT
63	HbSDG29	XM_021811847.1	TTCAGAAGCCATCACAGCAG	CCCAGGGTTCTTTTCCATCC
64	HbSDG30	XM_021807326.1	GGTGGTGCAATATTCAGGCT	TCCCCACCTCTTCATCAACT
65	HbSDG31	XM_021800564.1	AGATGGTCTCTCCTGCCTTT	TCTAAGGAGGCCACACTGTT
66	HbSDG32	XM_021794618.1	AACCATTCAGCTGTCCTTGG	CGTTTGAGTGTGATCCGTGA
67	HbSDG33	XM_021780908.1	TGCAGCATTGATGTAGGGAC	GCCAGCTTTCTCCACTCTAC
68	HbSDG34	XM_021810275.1	AGTGGAATAAAGGGCCTCCT	GTTACACTCCGCACATCAGT
69	HbSDG35	XM_021813550.1	GGTTAAACCCAGCAAGCCTA	TTTAGGAGAGGACACTGCCA
70	HbSDG36	XM_021810520.1	TTTGACCAGGAATGTGAGCC	AACTTCCTCAGCTTCTCCCT
71	HbSDG37	XM_021794939.1	AACGAAAGAGGCACAAGAGG	ATCCCTCTCATGCTCCTCAA
72	HbSDG38	XM_021825435.1	AGCTCAGCATGGGATTTGTT	CAGCAATGAGCACTTTGAGC
