# Supplementary material for: Identification of histone methylation modifiers and their expression patterns during somatic embryogenesis in Hevea brasiliensis
Source: Genet Mol Biol. 2020 Feb 17;43(1):e20180141. doi: 10.1590/1678-4685-GMB-2018-0141 (PMC7229888; doi:10.1590/1678-4685-GMB-2018-0141)
Supplement: Supplementary file 3 [file 1415-4757-GMB-43-1-e20180141-20200116-suppl3.pdf]

# Supplementary Material to "Identification of histone methylation modifiers and their expression patterns during somatic embryogenesis in *Hevea brasiliensis*"

**Table S3** - Overview of the detected histone modification genes in *Hevea brasiliensis*

| No. | Name | Accession number (GenBank) | Predicted gene length* | Predicted ORF length | Deduced polypeptide Length (aa) |
|---|---|---|---|---|---|
| 1 | HbLSD1 | XM_021786768.1 | 3142 | 2736 | 911 |
| 2 | HbLSD2 | XM_021834736.1 | 1801 | 1848 | 615 |
| 3 | HbLSD3 | XM_021834736.1 | 2445 | 2445 | 814 |
| 4 | HbLSD4 | XM_021828898.1 | 2384 | 2256 | 751 |
| 5 | HbLSD5 | XM_021806158.1 | 8063 | 6693 | 2230 |
| 6 | HbJMJ1 | XM_021782931.1 | 7163 | 3855 | 1284 |
| 7 | HbJMJ2 | XM_021803290.1 | 3967 | 1506 | 501 |
| 8 | HbJMJ3 | XM_021812039.1 | 8662 | 2631 | 876 |
| 9 | HbJMJ4 | XM_021824874.1 | 5902 | 2895 | 964 |
| 10 | HbJMJ5 | XM_021800084.1 | 9119 | 3933 | 1310 |
| 11 | HbJMJ6 | XM_021834835.1 | 13289 | 4824 | 1607 |
| 12 | HbJMJ7 | XM_021800066.1 | 6898 | 3567 | 1188 |
| 13 | HbJMJ8 | XM_021818539.1 | 7276 | 3090 | 1029 |
| 14 | HbJMJ9 | XM_021795250.1 | 4499 | 2358 | 785 |
| 15 | HbJMJ10 | XM_021821496.1 | 6340 | 2328 | 775 |
| 16 | HbJMJ11 | XM_021794700.1 | 4667 | 1353 | 450 |
| 17 | HbJMJ12 | XM_021786520.1 | 7568 | 3048 | 1015 |
| 18 | HbJMJ13 | XM_021827306.1 | 6805 | 3312 | 1103 |
| 19 | HbJMJ14 | XM_021817790.1 | 5757 | 2946 | 981 |
| 20 | HbJMJ15 | XM_021837061.1 | 13872 | 4353 | 1450 |
| 21 | HbJMJ16 | XM_021820973.1 | 4628 | 2634 | 877 |
| 22 | HbJMJ17 | XM_021807740.1 | 4419 | 2169 | 722 |
| 23 | HbJMJ18 | XM_021817272.1 | 7065 | 1884 | 627 |
| 24 | HbJMJ19 | XM_021817249.1 | 6341 | 3231 | 1076 |
| 25 | HbJMJ20 | XM_021817902.1 | 2862 | 1212 | 403 |
| 26 | HbPRMT1 | XM_021827573.1 | 4087 | 1791 | 596 |
| 27 | HbPRMT2 | XM_021827163.1 | 7618 | 1392 | 463 |
| 28 | HbPRMT3 | XM_021781563.1 | 4937 | 1140 | 379 |
| 29 | HbPRMT4 | XM_021787829.1 | 5127 | 2118 | 705 |
| 30 | HbPRMT5 | XM_021781563.1 | 3415 | 1617 | 538 |
| 31 | HbPRMT6 | XM_021792442.1 | 12133 | 1950 | 649 |
| 32 | HbPRMT7 | XM_021785572.1 | 1575 | 687 | 228 |
| 33 | HbPRMT8 | XM_021781562.1 | 3415 | 1239 | 412 |
| 34 | HbPRMT9 | XM_021790643.1 | 5325 | 1479 | 492 |
| 35 | HbSDG1 | XM_021783766.1 | 3514 | 2298 | 765 |
| 36 | HbSDG2 | XM_021798535.1 | 4797 | 2313 | 770 |
| 37 | HbSDG3 | XM_021814753.1 | 2019 | 2019 | 672 |
| 38 | HbSDG4 | XM_021779747.1 | 1761 | 1761 | 586 |
| 39 | HbSDG5 | XM_021789206.1 | 3425 | 1032 | 343 |

| No. | **Name** | **Accession number** | **Predicted gene** | **Predicted ORF** | **Deduced polypeptide** |
|---|---|---|---|---|---|
| | | **(GenBank)** | **length*** | **length** | **Length (aa)** |
| 40 | HbSDG6 | XM_021802565.1 | 2880 | 2439 | 812 |
| 41 | HbSDG7 | XM_021802564.1 | 3126 | 3126 | 1041 |
| 42 | HbSDG8 | XM_021790468.1 | 7856 | 4542 | 1513 |
| 43 | HbSDG9 | XM_021815283.1 | 7710 | 2778 | 925 |
| 44 | HbSDG10 | XM_021790438.1 | 8602 | 4368 | 1455 |
| 45 | HbSDG11 | XM_021789001.1 | 3062 | 1008 | 335 |
| 46 | HbSDG12 | XM_021795575.1 | 6222 | 2547 | 848 |
| 47 | HbSDG13 | XM_021812048.1 | 4918 | 2649 | 882 |
| 48 | HbSDG14 | XM_021790481.1 | 5703 | 2016 | 671 |
| 49 | HbSDG15 | XM_021835117.1 | 2342 | 1203 | 400 |
| 50 | HbSDG16 | XM_021784706.1 | 7516 | 1137 | 378 |
| 51 | HbSDG17 | XM_021803998.1 | 5391 | 972 | 323 |
| 52 | HbSDG18 | XM_021789490.1 | 1188 | 1188 | 395 |
| 53 | HbSDG19 | XM_021750304.1 | 4752 | 1272 | 423 |
| 54 | HbSDG20 | XM_021816041.1 | 7275 | 3843 | 1280 |
| 55 | HbSDG21 | XM_021829524.1 | 5650 | 2187 | 728 |
| 56 | HbSDG22 | XM_021790903.1 | 11265 | 3489 | 1162 |
| 57 | HbSDG23 | XM_021781966.1 | 8352 | 2655 | 884 |
| 58 | HbSDG24 | XM_021821775.1 | 8221 | 3222 | 1073 |
| 59 | HbSDG25 | XM_021809017.1 | 8205 | 2961 | 986 |
| 60 | HbSDG26 | XM_021832519.1 | 7288 | 3018 | 1005 |
| 61 | HbSDG27 | XM_021806151.1 | 2636 | 1044 | 347 |
| 62 | HbSDG28 | XM_021802187.1 | 3824 | 1206 | 401 |
| 63 | HbSDG29 | XM_021811847.1 | 14232 | 7539 | 2512 |
| 64 | HbSDG30 | XM_021807326.1 | 1632 | 1632 | 543 |
| 65 | HbSDG31 | XM_021800564.1 | 4010 | 1488 | 495 |
| 66 | HbSDG32 | XM_021794618.1 | 6088 | 1725 | 574 |
| 67 | HbSDG33 | XM_021780908.1 | 8834 | 4641 | 1546 |
| 68 | HbSDG34 | XM_021810275.1 | 15087 | 6510 | 2169 |
| 69 | HbSDG35 | XM_021813550.1 | 4111 | 822 | 273 |
| 70 | HbSDG36 | XM_021810520.1 | 7535 | 1539 | 512 |
| 71 | HbSDG37 | XM_021794939.1 | 5526 | 1581 | 526 |
| 72 | HbSDG38 | XM_021825435.1 | 3165 | 1992 | 663 |

Note:

* Include intron length
